# Effects of pomegranate extract on preventing dental caries: a systematic review

**DOI:** 10.3389/froh.2025.1484364

**Published:** 2025-05-27

**Authors:** Niyousha Rafeie, Yasaman Salimi, Zahra Sadat Aghamir, Amirhesam Amini, Hamed Taheri, Sarvin Sadreddini, Fatemeh Kamali, Golnesa Akbarian, Nazanin Azizi, Mobina Bagherianlemraski, Maryam Valizadeh, Farnoosh Alimohammadi, Negar Sedighnia, Mohammad Qadirifard, Mahdyieh Naziri

**Affiliations:** ^1^ Department of Biomaterials and Biomimetics, Division of Biomaterials, College of Dentistry, New York University, New York, NY, United States; ^2^ Department of Periodontics, School of Dentistry, Guilan University of Medical Sciences, Rasht, Iran; ^3^ Department of Dental Research, School of Dentistry, Tehran University of Medical Sciences, Tehran, Iran; ^4^ Department of Restorative Dentistry, School of Dental Medicine, University of Pennsylvania, Philadelphia, PA, United States; ^5^ Department of Dentistry and Implantology, Institute of Fundamental Medicine and Biology, Kazan (Volga Region) Federal University, Russia; ^6^ Department of Dental Research, Student Research Committee, Tabriz University of Medical Sciences, Tabriz, Iran; ^7^ Department of Research, Faculty of Pharmacy, Tabriz University of Medical Sciences, Tabriz, Iran; ^8^ Department of Health, Research Methods, Evidence, and Impact, McMaster University, Hamilton, ON, Canada; ^9^ Department of Dental Research, Dental Research Center, Mashhad University of Medical Sciences, Mashhad, Iran; ^10^ Department of Dental Research, School of Dentistry, Shahid Beheshti University of Medical Sciences, Tehran, Iran; ^11^ Department of Dental Research, Faculty of Dentistry, Tabriz University of Medical Sciences, Tabriz, Iran; ^12^ Department of Prosthodontics, Faculty of Dentistry, Babol University of Medical Sciences, Babol, Iran; ^13^ Department of Health, School of Health, Iran University of Medical Science, Tehran, Iran

**Keywords:** dental caries, pomegranate, *Punica granatum*, *Streptococcus mutans*, tooth demineralization

## Abstract

**Background/purpose:**

The present study aimed to evaluate the effect of pomegranate extract on the prevention of dental caries compared to standard care, placebo, and no intervention.

**Materials and methods:**

A bibliographic search in four databases, including PubMed, Scopus, Google Scholar, and CENTRAL, yielded 291 studies until September 8, 2023. The search was performed among the studies written in English using the search terms “*Punica granatum*” AND (“dental caries” OR “*Streptococcus mutans*” OR “tooth demineralization”) After screening the titles/abstracts and full texts of these studies, 7 articles were chosen.

**Results:**

In all 7 articles, pomegranate mouthwash was used as the intervention. In 5 studies, the control group used 0.2% chlorhexidine (CHX) mouthwash. Additionally, 4 articles reported a reduction in the mean *Streptococcus mutans* plaque count in both groups; however, better results were observed in the CHX mouthwash group. In one study, no significant difference was reported between the study and control groups. Finally, one study showed the significant superiority of a hydroalcoholic extract of pomegranate mouthwash over CHX mouthwash.

**Conclusion:**

Overall, the results suggest that pomegranate extract mouthwash is highly effective in reducing caries-causing bacteria. No side effects were reported for pomegranate use in these studies.

**Systematic Review Registration:**

https://osf.io/69gpc/

## Introduction

Although the prevalence of dental caries has decreased in recent years, it is still considered among the most common chronic diseases in both adults and children (1, 2). Globally an estimated 3 billion people suffer from dental caries, with 60%–90% of adults and children experiencing it during their lifetime (3). In this regard, Poor oral health has been linked to systemic conditions, including neurovascular and cardiovascular diseases, due to chronic inflammation and bacterial infiltration from carious lesions (4–7).

Many factors have been associated with initiating dental caries, including poor oral hygiene, xerostomia, a high-sugar diet, fluoride deficiency, and inadequate routine dental care (8). Among these, bacterial colonization is considered the primary driver of caries incidence (1, 2). *Streptococcus mutans* (*S. mutans*), in particular, plays a key role in caries initiation by adhering to tooth surfaces and forming plaque biofilm (9). Alongside other bacteria, *S. mutans* ferments dietary carbohydrates, producing acids that demineralize enamel and dentin, leading to mineral loss and cavitation (10–12). Additionally, *S. mutans* enhances biofilm formation by producing extracellular polysaccharides, which provide adhesion sites and nutrients for other bacteria (13).

Various preventive strategies have been proposed to reduce dental caries, such as limiting sugar intake, water fluoridation, improving oral hygiene, regular dental visits, fissure sealants, and silver diamine fluoride application (3, 14–16). Medicinal plant extracts have also gained attention for their potential to inhibit biofilm formation by either preventing bacterial adhesion or reducing cariogenic bacterial counts (17). However, few natural compounds have been widely adopted due to limitations in taste, cost, efficacy, odor, and stability (18).

Among natural agents, pomegranate (*Punica granatum* L.) has been extensively studied for its antimicrobial properties. Traditionally used to treat dysentery, diarrhea, and respiratory infections (19, 20). pomegranate extracts have demonstrated efficacy against bacterial and viral pathogens (13, 21–23). Notably, pomegranate aril and peel extracts exhibit antimicrobial activity against *Staphylococcus aureus* and *Escherichia coli* (24). The fruit's phytochemicals, particularly ellagic acid and hydrolyzable tannins (e.g., punicalagin), contribute to its antimicrobial effects (25).

Recent studies have demonstrated that pomegranate peel polyphenolic extracts are effective against both planktonic and biofilm-forming oral bacteria such as *S. mutans*, *S. mitis*, *S. oralis*, and *R. dentocariosa* (1, 26). Synergistic effects with agents like myrtle or honey further enhance biofilm disruption through mechanisms such as adhesion inhibition, interference with matrix synthesis, and membrane destabilization (27, 28).

Antimicrobial peptides derived from pomegranate have also shown anti-cariogenic effects by blocking *S. mutans* adhesion without toxicity to human keratinocytes (29). Other studies support pomegranate's broad-spectrum activity against cariogenic bacteria (1, 30–32).

Despite promising evidence, a comprehensive systematic review is needed to unite findings and guide clinical applications. This study aims to evaluate the efficacy of pomegranate extract in preventing dental caries compared to standard care, placebo, or no intervention.

## Materials and methods

This systematic review was conducted in accordance with the PRISMA (Preferred Reporting Items for Systematic Reviews and Meta-Analyses) guidelines (33) to ensure methodological rigor and transparency. A pre-registered protocol titled “*Effects of Pomegranate Extract on Preventing Dental Caries: A Systematic Review”* was filed with the Open Science Framework (OSF; registration DOI: 10.17605/OSF.IO/69GPC and URL: https://osf.io/69gpc/).

### Eligibility criteria

#### Inclusion criteria

This study included randomized clinical trials (RCTs) involving healthy participants of any age classified as high-risk for dental caries based on validated caries risk assessment tools.

#### Exclusion criteria

Exclusion criteria comprised individuals currently using mouthwash or who had taken medications that reduce salivary flow (such as antibiotics or anti-inflammatory drugs) within the past month. We also excluded current or former alcohol consumers, smokers, and users of paan or gutka. Medically compromised individuals (e.g., those with diabetes mellitus, renal disease, gastrointestinal disorders, or respiratory diseases) were excluded, as were patients undergoing orthodontic treatment, dental rehabilitation, or those with missing teeth (unless due to reasons unrelated to dental caries). Additional exclusions included individuals with a history of radiation therapy, active oral infections (e.g., abscess, cellulitis), or conditions requiring pulp therapy or extraction, as well as those with known allergies to mouthwash or study-related drugs. Non-randomized studies, animal research, case reports, case series, letters to the editor, and gray literature were also excluded.

### Data extraction

Two independent reviewers conducted a systematic search of English-language articles published in peer-reviewed journals between 2006 and 2023. The search was performed from December 2, 2018, to September 8, 2023, across PubMed, Scopus, and Google Scholar. Search terms included “*Punica granatum”* [MeSH], “*dental caries”* [MeSH], “*Streptococcus mutans”* [MeSH], and “*tooth demineralization”* [MeSH]. The initial search results were compiled into an abstract database focusing on studies examining the effects of *Punica granatum* on dental caries. The search strategy outcomes are summarized in Table 1.

**Table 1:** Search strategy output used in the present study for data extraction.

Search engine	Search strategy	Additional filter
PubMed/Medline	((“Pomegranate” [Mesh]) OR (“*Punica granatum*” [Mesh])) AND ((“Dental Caries” [Mesh]) OR (“*Streptococcus mutans*”[Mesh]) OR (“tooth demineralization”[Mesh])) OR ((Pomegranate[Title/Abstract]) OR (*Punica granatum*[Title/Abstract])) AND ((dental caries[Title/Abstract]) OR (*streptococcus mutans*[Title/Abstract]) OR (tooth demineralization[Title/Abstract]))	English, Aguste 8th, 2022
Scopus	((TITLE-ABS-KEY (dental AND caries) OR TITLE-ABS-KEY (*streptococcus* AND *mutans*) OR TITLE-ABS-KEY (tooth AND demineralization))) AND ((TITLE-ABS-KEY (pomegranate*) OR TITLE-ABS-KEY (*punica* AND *granatum*)))	English, Aguste 8th, 2022
Central	#1: (“dental caries”):ti,ab,kw OR (dental):ti,ab,kw OR (caries):ti,ab,kw OR (tooth decay):ti,ab,kw OR (*streptococcus mutans*):ti,ab,kw (Word variations have been searched)	English, Aguste 8th, 2022
	#2: (“*mutans streptococci*”):ti,ab,kw OR (“*mutans streptococcus*”):ti,ab,kw OR (*mutans*):ti,ab,kw OR (tooth demineralization):ti,ab,kw (Word variations have been searched)	
	#3: MeSH descriptor: [Tooth Demineralization] explode all trees	
	#4: MeSH descriptor: [Dental Caries] explode all trees	
	#5: MeSH descriptor: [*Streptococcus mutans*] explode all trees	
	#6: (“*Punica granatum*”):ti,ab,kw OR (“pomegranate”):ti,ab,kw	
	#7: MeSH descriptor: [Pomegranate] explode all trees	
	#8: (#1 OR #2 OR #3 OR #4 OR #5) AND (#6 OR #7)	

### Quality assessment

fter the initial literature search, abstracts were screened for consistency with the PICOS (Population, Intervention, Comparison, Outcomes, Study design) objectives. Studies that did not address the primary research questions of this systematic review were excluded.

Articles selected for full-text review were entered into an Excel spreadsheet, and the two reviewers assessed agreement on inclusion. The final selection was based on a full-text review, with study quality and risk of bias evaluated using the Cochrane Collaboration's tool.

This study prioritized *in vitro* and *in vivo* research using dental models (human or bovine tooth fragments) and interventions involving *Punica granatum* extract. Additional relevant studies were identified through snowball sampling (citation tracking of reference lists in selected articles) and included if they met the primary and secondary search criteria.

Following the final review, the reviewers summarized the findings, identified key themes, and reached a consensus on the reported outcomes.

## Results

### Study selection

**Figure 1:**
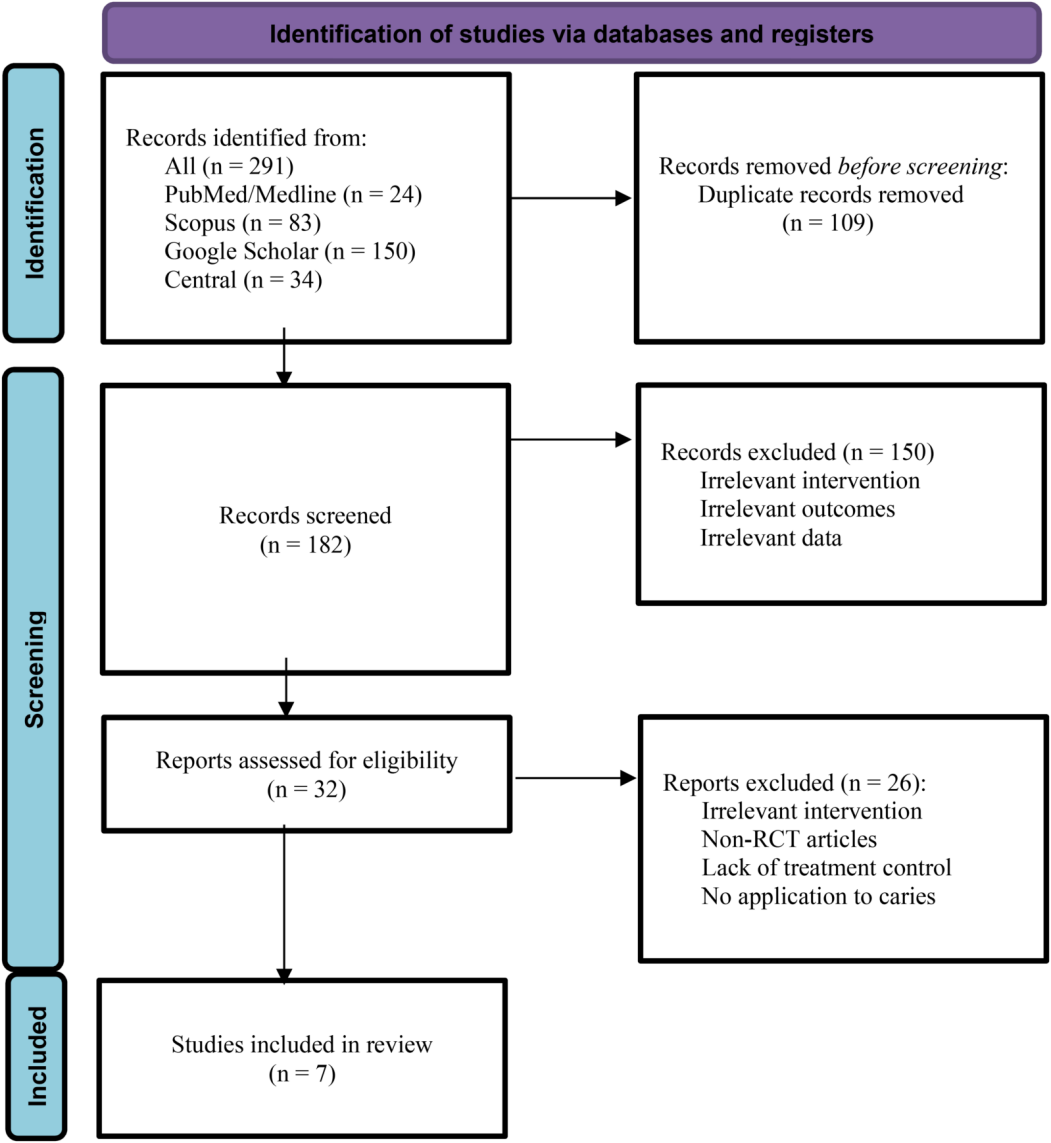
PRISMA flowchart used for detailing the database searches, the number of abstracts screened, and the full texts retrieved.

The results of the evaluated studies are summarized in Table 2. Initial database searches using predefined keywords yielded 83 articles from Scopus, 24 from PubMed, 150 from Google Scholar, and 31 from the CENTRAL (Cochrane Central Register of Controlled Trials) database. After removing duplicates using EndNote X9, 182 articles remained. Two reviewers independently conducted title/abstract and full-text screening. Ultimately, six articles that evaluated the effect of *Punica granatum* on dental caries met the inclusion criteria (Figure 1). The included studies assessed outcomes such as tooth demineralization, *Streptococcus mutans* count, and dental caries.

**Table 2:** Data of the included studies on the effect of pomegranate on dental caries.

First author (Year)	Country	Type of study	Follow-up duration	Intervention	Participants	Control group	Outcome
Kadam et al. (38)	India	RCT	Salivary samples were collected at baseline and at 10, 30, and 60 min after mouthwash use.	3 ml of pomegranate peel extract rinse for 2 min. Peel sun-dried, powdered, then aqueous extract prepared.	30 children (6–8 Y/O), divided into two groups of 15 individuals	10 ml of 0.2% CHX mouthrinse for 2 min	• Increased salivary pH • Lowered saliva activities of alpha-glucosidase • Increased ceruloplasmin activity
Srilekha et al. (34)	India	Single-blind RCT	Plaque samples collected at baseline and after 7 days of follow-up.	15 ml of commercial *P. granatum* mouthwash for 30 s.	20 dental students, divided into two groups of 10	15 ml of 0.2% chlorhexidine mouthwash for 30 s	• Reduced *S. mutans* count • No significant difference between chlorhexidine and *P. granatum* in reducing *S. mutans*
Bansal et al. (37)	India	RCT	Salivary samples collected at baseline, day 7 after thorough scaling and polishing, and daily use of mouthwashes.	140 ml of 15% *P. granatum* mouthwash daily. Likely derived from peel.	60 children (8–12 Y/O), divided into 3 groups of 20	Group A: 0.2% chlorhexidine mouthwash Group B: 10% *Achyranthes aspera* mouthwash	• Reduced plaque index • Reduced *S. mutans* count • Chlorhexidine marginally more effective than *P. granatum* against *S. mutans*
Mishra et al. (35)	India	Double-blind RCT	Salivary samples collected at baseline, days 16, and 31.	10 ml of *P. granatum* mouthwash daily after dinner.	80 children (8–15 Y/O), divided into 4 groups of 20	Group B: *Terminalia chebula* Group C: *Vitis vinifera* Group D: 0.2% chlorhexidine mouthrinse	• Decreased plaque score at day 16 • Lowest plaque score at day 31 • No significant difference in buffering capacity • Reduced *S. mutans* count, maximum reduction in *P. granatum* group
El Naggar et al. (40)	Egypt	RCT	Salivary samples collected at baseline, immediately after mouthwash usage, and after 7 days.	Pomegranate extract mouthwash (MIC = 3.9 mg/ml), used 3x/day. Extracted from whole fruit (arils, seeds, peel) using ethanol + distilled water.	80 participants (17–50 Y/O), divided into 4 groups of 20	Group 1: Propolis mouthwash (MIC = 1.172 mg/ml) Group 3: Chlorhexidine mouthwash Group 4: Saline	• Reduced *S. mutans* count • Similar antibacterial effect of pomegranate and chlorhexidine • Least effect on taste alteration compared to chlorhexidine and saline
Menezes et al. (39)	Brazil	RCT	Plaque samples collected at baseline and after 1 min of mouthwash use (no brushing for 24 h).	Hydroalcoholic extract of *P. granatum* mouthrinse (50–60 mg/ml).	60 patients (9–25 Y/O) with fixed orthodontic appliances, divided into 3 groups of 20	Group 2: 12% chlorhexidine gluconate mouthwash Group 3: Distilled water (negative control)	• Similar antibacterial activity to chlorhexidine against several pathogens • No efficacy against *Pseudomonas*, unlike chlorhexidine
Ibrahim et al. (36)	Egypt	Interventional RCT	Plaque samples collected 30 min after rinsing and incubated for 48–96 h.	Pomegranate extract (arils + seeds) and peel, separately extracted and prepared with water.	84 children (5–8 Y/O) with no systemic disease or recent antibiotic use, divided into 4 groups	Group 1: Chlorhexidine mouthwash	• Significant reduction in *S. mutans* count (*p* < 0.001) • *P. granatum* mouthwash showed greatest reduction in bacterial count

RCT, randomized controlled trial; *S. mutans, Streptococcus mutans*; CHX, chlorhexidine, MIC, inimum nhibitory oncentration, ADA, American dental association), DMFT, decayed, missing, and filled teeth); Y/O, years old.

### Risk of bias assessment

**Figure 2:**
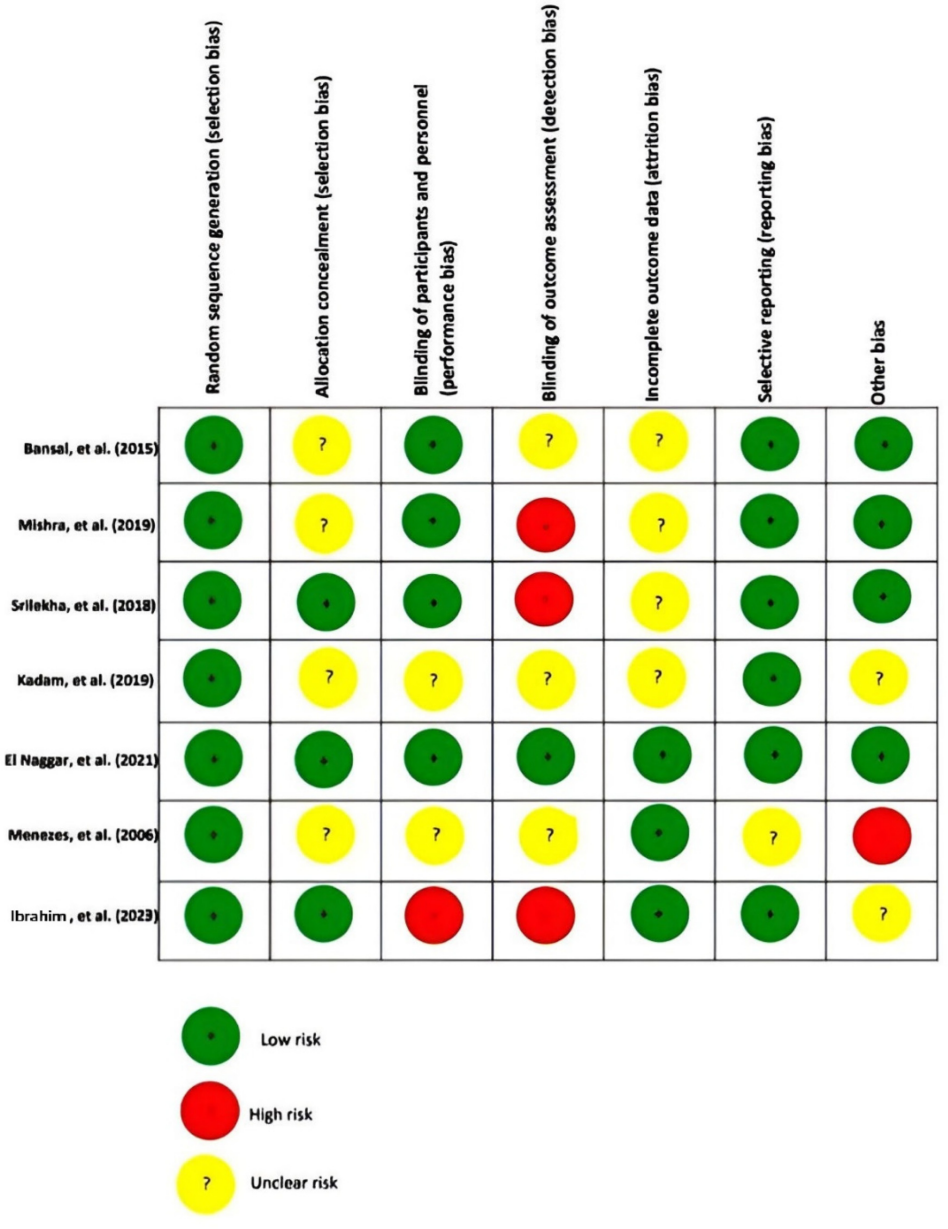
Quality assessment of the articles included in the present study.

The risk of bias in this review article was examined using the Cochrane Risk of Bias assessment tool. Three of these articles had a high-risk bias for criteria of detection bias (34–36), and 3 had an unclear risk for selection bias (35, 37, 38). Four of them had an unclear risk for attrition bias (34, 35, 37, 38), and two had an unclear risk for performance bias (36, 37). Figure 2 summarizes the evaluation bias risk of the included articles.

### Study locations and design

Among seven articles, 4 were performed in India (34, 35, 37, 38), 1 in Brazil (39), and 2 in Egypt (36, 40). All of the studies were randomized clinical trials (34–40).

### Study participants

In four of them, participants were children (35–37), and in 2 articles, adults were evaluated (34, 35, 37–40). Finally, 1 article included participants with a wide age range from 9 to 50 years (39).

### Preparation methods of pomegranate extracts

All seven studies used pomegranate mouthwash as the intervention. Preparation methods varied by plant part (peel, juice, or whole fruit), solvent, and technique. Peel extracts were most common. Kadam et al. (38) sun- and oven-dried the peel, powdered it, and used aqueous Soxhlet extraction. Bansal et al. (37) and Mishra et al. (35) also used peel extracts but did not describe methods. Whole fruit extracts were used by El Naggar et al. (40) and Menezes et al. (39) Juice and peel extracts were used by Ibrahim et al. (36), juice was hand-extracted and concentrated by heating; peel was air-dried, pulverized, mixed with water, and filtered. Srilekha et al. (34) used a commercial product with unspecified extraction details.

### Control interventions

In six studies, control groups used 0.2% chlorhexidine (CHX) mouthwash (34–38, 40). Among these, three also included other mouthwashes in their experimental groups: Mishra et al. (35) used *Terminalia chebula* and *Vitis vinifera* extracts; Bansal et al. (37) included 10% *Achyranthes aspera*; and El Naggar et al. (40) evaluated saline alongside CHX. Only one study did not use CHX; instead, the control group received distilled water (39).

### Antibacterial and clinical outcomes

Seven studies evaluated the effects of *Punica granatum* (pomegranate) mouthwash on oral health outcomes, including antibacterial efficacy, salivary pH, enzymatic activity, and plaque index. Five studies (34–37, 39) reported a reduction in *S. mutans* count following pomegranate mouthwash use. In Bansal et al. (37), *P. granatum*, CHX, and *A. aspera* mouthwashes significantly reduced both plaque index and *S. mutans* count after seven days, though CHX showed the greatest effect. Mishra et al. (35) found pomegranate mouthwash produced the largest decrease in *S. mutans* compared to *T. chebula*, *V. vinifera*, and CHX, although pH and buffering capacity did not differ significantly between groups. Their findings suggest *P. granatum*'s preventive effect may involve inhibiting hydrolytic enzymes or interfering with bacterial adhesion to teeth.

El Naggar et al. (40) compared the effects of saline (control), CHX, pomegranate, and propolis mouthwashes. All groups showed an immediate rise in salivary pH post-use, with no significant differences between groups. After seven days, pH significantly declined in the pomegranate and propolis groups, while no change was observed in the CHX and saline groups. Bacterial counts were similar across all groups. This study noted that pomegranate mouthwash reduced alpha-glucosidase activity and increased ceruloplasmin, an antioxidant enzyme.

Menezes et al. (39) demonstrated that a hydroalcoholic pomegranate extract exhibited strong antibacterial activity against dental plaque microorganisms. It reduced CFU/ml by 84%, outperforming CHX (79%) and distilled water (11%). Similarly, Ibrahim et al. (36) found that pomegranate mouthwash led to a significant reduction in *S. mutans* count in children, with the greatest antibacterial effect observed in the pomegranate group compared to other treatments, such as CHX.

Finally, Srilekha et al. (34) assessed *S. mutans* counts after immediate and seven-day use of CHX and pomegranate mouthwash. Both groups showed significant reductions at day seven compared to baseline, with no significant difference between them. They also reported that pomegranate's effects were linked to reduced alpha-glucosidase activity and enhanced ceruloplasmin levels. Participants in this study had fair-to-good plaque index scores. Kadam et al. (38) also reported that pomegranate peel extract mouthwash significantly increased salivary pH and decreased salivary alpha-glucosidase activity, an enzyme that breaks down sucrose, while also enhancing ceruloplasmin activity, an antioxidant enzyme. The authors concluded that pomegranate mouthwash may have protective effects against dental caries by influencing these enzymatic activities.

## Discussion

This is the first systematic review to comprehensively examine the effects of *Punica granatum* mouthwash on salivary pH, *S. mutans* levels in saliva and dental plaque, and plaque index.

### Adverse effects of CHX vs. *Punica granatum* mouthwash

CHX mouthwash is commonly prescribed as the gold standard for preventing plaque formation and gingivitis. However, long-term use of CHX is associated with several side effects. Commonly reported effects include taste alteration, oral numbness, dry mouth (xerostomia), and discoloration of the tongue and teeth, with tooth staining being the most frequent reason for discontinuation (41). Other long-term effects may include calculus accumulation, oral paraesthesia, mucosal desquamation, and, in rare cases, parotid gland swelling (42). Additionally, concerns have arisen regarding antimicrobial resistance, particularly with ESKAPE pathogens, following repeated low-level exposure to CHX (43).

*Punica granatum* mouthwash did not cause taste changes compared to CHX (40). None of the studies reported adverse events associated with *Punica granatum* mouthwash. A potential limitation in the reviewed studies is the inadequate reporting of adverse events. Most trials did not specify whether adverse effects were actively monitored, and none used standardized tools for adverse event assessment. Short follow-up durations, especially in studies with outcomes measured within minutes to a few days, may have further limited the detection of delayed or mild side effects.

### Polyphenolic components and their antimicrobial action in *Punica granatum* extract

The antimicrobial effect of *P. granatum* is primarily attributed to its polyphenols. These polyphenols may affect bacterial cell walls, discourage microbial coaggregation, and inhibit bacterial enzymes through oxidation (44). The bioactivity of pomegranate extract is largely due to its high content of ellagitannins, particularly punicalagin. Punicalagin modulates oxidative stress by scavenging free radicals and inducing cellular antioxidant defenses, such as upregulation of superoxide dismutase and glutathione peroxidase, while suppressing pro-inflammatory cytokines like IL-6 and TNF-α (45, 46). A study by Scaglione et al. (47) found that pomegranate peel extract (PPE) exhibited antimicrobial activities without inducing ROS production or cytotoxicity in human cell lines after 30 h, supporting its selective bioactivity and low toxicity in primary cells and macrophage models (48). Furthermore, the depletion of punicalagin and related compounds during bacterial incubation, as observed by Peppoloni et al. (49) suggests active interaction with microbial targets, likely through membrane disruption, enzyme inhibition, and interference with quorum sensing, as proposed in studies of polyphenol–pathogen dynamics (50).

### Microbial composition of dental plaque and effects of *Punica granatum* on Non-*S. mutans* Species

In addition to *S. mutans*, dental plaque harbors over 400 distinct bacterial species that digest carbohydrates, produce acid, and contribute to the development of dental caries. These microorganisms can be broadly categorized based on oxygen tolerance, Gram staining characteristics, and morphology (51):

• Gram-positive aerobes: *S. sanguis, S. sobrinus, S. salivarius, Actinomyces viscosus*
• Gram-negative facultatives: *Actinobacillus, Capnocytophaga, Eikenella corrodens*
• Gram-negative anaerobes: *Porphyromonas gingivalis, Fusobacterium nucleatum, Prevotella intermedia, Bacteroides forsythus, Campylobacter rectus*
• Spirochetes: *Treponema denticola*

Among the reviewed studies, only one evaluated the antimicrobial activity of *Punica granatum* mouthwash and CHX against non-*Streptococcus mutans* species in dental plaque. Both agents demonstrated efficacy against opportunistic pathogens, including *Staphylococcus* spp., *Escherichia coli*, *Klebsiella* spp., and *Proteus* spp. Notably, *Staphylococcus epidermidis* was found to be susceptible to the hydroalcoholic extract (HAE) of *Punica granatum* but resistant to CHX (39).

### Effectiveness of *Punica granatum* mouthwash in modifying salivary pH

Saliva's pH and buffering capacity are critical in determining the risk of dental caries. Acidic pH promotes enamel demineralization, increasing the risk of caries (52). This review found that *Punica granatum* mouthwash increases salivary pH.

However, the comparative effectiveness of *Punica granatum* relative to chlorhexidine (CHX) remains inconclusive. In a study by El Naggar et al. (40), CHX use over 7 days resulted in a significantly greater increase in salivary pH compared to *Punica granatum* mouthwash, indicating superior efficacy. In contrast, findings from Mishra et al. (35) and Kadam et al. (38) showed no statistically significant difference between the two interventions, suggesting comparable outcomes.

The time frame in which *Punica granatum* mouthwash affects salivary pH varies widely across studies, ranging from immediate effects to 15 days. Even a single use of *Punica granatum* mouthwash has been shown to significantly increase salivary pH (40). However, comparisons between studies are difficult due to differences in factors such as mouthwash concentration, formulation type (aqueous or hydroalcoholic), and frequency of use. Therefore, while *Punica granatum* shows promise in increasing salivary pH, further studies are needed to determine whether it consistently matches the effectiveness of CHX.

### Effect of *Punica granatum* mouthwash on plaque formation and bacterial reduction

Furthermore, only two studies explicitly showed the *Punica granatum* mouthwash decreased the plaque index with the same efficacy as CHX (35, 37). In terms of *S. mutans* reduction in dental plaque, theree of the included studies reported similar antimicrobial efficacy between *P. granatum* and CHX (34, 39, 40) although the aqueous extract used in Menezes et al. (39), was less effective than the hydroalcoholic version and CHX. One study found CHX to be marginally more effective than *P. granatum* (37), while Ibrahim et al. (36) reported greater bacterial reduction with *P. granatum* than CHX.

It has been suggested that *P. granatum* reduces dental plaque by inhibiting the bacteria responsible for its formation. Tannins such as punicalin and punicafolin, found in pomegranate peel, inhibit human salivary alpha-amylase, an enzyme that facilitates plaque formation by providing substrates for cariogenic bacteria. Alpha-amylase catalyzes the hydrolysis of starch to produce oligosaccharides, which bind to both enamel and cariogenic bacteria, promoting plaque formation. By preventing bacterial adhesion to enamel, *P. granatum* helps reduce plaque formation (53, 54).

### Demographics and sample characteristics of included studies

Most of the reviewed studies involved healthy children and young adults aged 6–15 years. Only two studies assessed the efficacy of *Punica granatum* mouthwash in individuals at high caries risk: one focused on 9–25-year-olds using fixed orthodontic appliances (39), and the other included 17–50-year-olds with high caries risk based on the ADA caries risk assessment (40).

The studies used various parts of *Punica granatum*, including seeds (38), fruit (34), peel (37), arils, exocarp, and mesocarp (40). Only one study extracted a hydroalcoholic solution directly from *Punica granatum* and evaluated its effect on *S. mutans* levels in dental plaque (39). Therefore, it remains unclear which specific component of *Punica granatum* is responsible for its antibacterial effects against *S. mutans*.

### Summary and limitations of *Punica granatum* mouthwash in oral health

Compared to a previous systematic review evaluating the efficacy of pomegranate-based products in oral health, the systematic review and meta-analysis by Javan et al. (55) examined the effects of pomegranate vs. CHX on periodontal indices such as plaque index, gingival index (GI), and bleeding index (BI). Their results showed that while CHX had a significantly greater impact on plaque reduction at 14–15 days, no significant differences were found in shorter durations or for GI and BI, suggesting comparable anti-inflammatory effects between *Punica granatum* and CHX in periodontal contexts. similarly in our review, while most included studies also reported CHX to be more effective, at least one study demonstrated superior efficacy of hydroalcoholic pomegranate extract, and none reported adverse effects with *Punica granatum* use. Thus, both reviews highlight the antimicrobial and anti-inflammatory potential of pomegranate. In conclusion, our findings from the reviewed studies showed that *Punica granatum* mouthwash has antimicrobial potential compared to CHX mouthwash without any reported side effects. This mouthwash might be beneficial in preventing dental caries by decreasing the plaque index, increasing salivary pH, and reducing the levels of S. mutans in saliva and dental plaques. Such mouthwash would be less costly and well tolerated by patients.

Nevertheless, this systematic review has several limitations that should be taken into account when interpreting the results. First, the number of available studies on *Punica granatum* mouthwash was limited, and most had relatively small sample sizes. There was also considerable variation among the studies in terms of the type of formulation used (aqueous vs. hydroalcoholic), the concentration of *P. granatum* extract, the duration and frequency of use, and the specific plant parts used (peel, seeds, fruit, or whole fruit components). Additionally, only a few studies investigated the antimicrobial activity of *P. granatum* against a broader range of oral bacteria beyond *S. mutans*, despite the fact that dental plaque contains a diverse bacterial population. The follow-up periods were generally short—ranging from a single application to just a few days—which limits the understanding of the mouthwash's long-term effectiveness and safety. While no adverse effects were reported, most studies did not clearly describe how adverse events were monitored, raising the possibility of underreporting. These limitations highlight the need for larger, well-designed clinical trials using standardized mouthwash formulations, longer follow-up durations, broader microbial assessments, and more rigorous safety reporting.

## Data Availability

The original contributions presented in the study are included in the article/Supplementary Material, further inquiries can be directed to the corresponding authors.
